# Direct quantification of skeletal pneumaticity illuminates ecological drivers of a key avian trait

**DOI:** 10.1098/rspb.2023.0160

**Published:** 2023-03-29

**Authors:** Maria Grace P. Burton, Roger B. J. Benson, Daniel J. Field

**Affiliations:** ^1^ Department of Earth Sciences, University of Cambridge, Downing Street, Cambridge CB2 3EQ, UK; ^2^ Department of Earth Sciences, University of Oxford, South Parks Road, Oxford OX1 3AN, UK; ^3^ Museum of Zoology, University of Cambridge, Downing Street, Cambridge CB2 3EJ, UK

**Keywords:** skeletal pneumaticity, air-filled bones, ecological adaptation, body mass, skeletal architecture, humerus

## Abstract

Skeletal pneumaticity is a key feature of extant avian structure and biology, which first evolved among the non-flying archosaurian ancestors of birds. The widespread presence of air-filled bones across the postcranial skeleton is unique to birds among living vertebrates, but the true extent of skeletal pneumaticity has never been quantitatively investigated—hindering fundamental insights into the evolution of this key avian feature. Here, we use microCT scans of fresh, frozen birds to directly quantify the fraction of humerus volume occupied by air across a phylogenetically diverse taxon sample to test longstanding hypotheses regarding the evolution and function of avian skeletal pneumatization. Among other insights, we document weak positive allometry of internal air volume with humeral size among pneumatized humeri and provide strong support that humeral size, body mass, aquatic diving, and the presence or absence of pneumaticity all have independent effects on cortical bone thickness. Our quantitative evaluation of humeral pneumaticity across extant avian phylogeny sheds new light on the evolution and ontogenetic progression of an important aspect of avian skeletal architecture, and suggests that the last common ancestor of crown birds possessed a highly pneumatized humerus.

## 1. Background

Skeletal pneumaticity refers to the presence of air-filled cavities within bones. Although pneumaticity of the skull is common among archosaurs and mammals [1–3], postcranial skeletal pneumaticity (PSP) is unique to birds among extant tetrapods (e.g. [4]), making it one of the most distinctive features of the avian skeleton. The origins of avian PSP can be traced back to the Late Triassic (approx. 210 Ma), with osteological correlates of PSP observed in several groups of extinct ornithodiran (bird-line) archosaurs, such as pterosaurs, sauropodomorph dinosaurs, non-avian theropods and Mesozoic avialans (e.g. [5–16]). Postcranial skeletal pneumatization in birds is enabled by the unique, structurally heterogeneous structure of the avian respiratory system—birds have dorsally immobilized lungs that are specialized for gas exchange and are ventilated by a number of highly compliant air sacs (usually nine) that are not significantly involved in gas exchange. The mechanism by which bones of the postcranial skeleton become pneumatized is understood to take place during post-hatching development through the growth of pneumatic diverticula, which are epithelial extensions from the pulmonary air sac system [4,10,17–25].

Although substantial progress has been made towards understanding the nature of PSP in birds, major gaps in our knowledge remain. Indeed, numerous factors are probably involved in determining the extent of PSP throughout the avian skeleton, such as body size, ecology and the biomechanical loading environment of individual skeletal elements (e.g. [3,14,20,26–32]). However, these links remain to be robustly tested across the avian crown group as a result of challenges inherent to quantifying the extent of avian skeletal pneumaticity.

As a step towards addressing these major gaps in our knowledge, we present a methodological approach using fresh, frozen birds to quantify the fractions of avian bones (in this case the humerus) occupied by air, marrow and bone. Although aspects of PSP have been investigated previously using microCT scans of fresh birds with intact soft tissues (e.g. [33,34]), this study is the first to explicitly quantify the extent of pneumaticity of specific skeletal elements and to visualize *in situ* soft and hard tissues within pneumatized long bones. We focus on the humerus, as it is the most frequently pneumatized long bone in the skeleton of extant birds [20,26,35].

Our data allow us to document macroevolutionary patterns in PSP across the avian tree of life and test several existing hypotheses regarding evolutionary acquisitions and losses of PSP. Among other hypotheses, we address whether the previously reported positive, though weak, correlation between body size and extent of pneumaticity (expressed as the total number of pneumatized postcranial skeletal elements; [20,26,29,30,35]), is mirrored by a positive association between body size and extent of pneumaticity within individual skeletal elements (expressed as the fraction of total bone volume occupied by air). Previous studies also suggest associations between indices of skeletal pneumaticity and certain ecological traits, especially aspects of foraging and locomotion (e.g. a general increase of pneumaticity in static soaring taxa and a general decrease of pneumaticity in aquatic diving taxa). Here, we focus specifically on assessing the hypothesis that aquatic diving taxa exhibit increased skeletal density, not just through reduction in the extent of bone volume occupied by air, but also through an increase in bone thickness, which has been interpreted as an energy-saving adaptation for maintaining neutral buoyancy underwater [31,36–38].

## 2. Methods

### (a) Taxon sampling

Whereas studies of avian PSP have generally restricted their investigations to focal neornithine subclades (e.g. [20,26–29,35]), we investigated a phylogenetically diverse sample of extant birds, spanning the avian crown group. Representatives of 20 ordinal-level clades and a total of 60 extant species were sourced from the University of Cambridge Museum of Zoology (UMZC), and, in the case of our *Eudromia elegans* specimen, the Royal Veterinary College (RVC) (see electronic supplementary material, tables S1 and S2 for specimen information). Sampled specimens were intact, freshly frozen adult individuals of both sexes. Since no specimens were collected specifically for this study, our taxon sample relied on the availability of frozen whole birds in accessible museum collections; as such, it was challenging to fully control for the level of decay and time between death and freezing for every specimen. To attempt to control for these factors, we visually assessed the extent of specimen decomposition and damage due to freezing degradation that could lead to soft-tissue volume loss, and excluded specimens exhibiting clear evidence of decomposition or damage. We calculated average species values for individuals in which both humeri were investigated, and for species in which multiple individuals were sampled.

### (b) Obtaining volumetric data

Whole frozen birds were microCT scanned without thawing using a Nikon XTEK H 225 ST scanner at the Cambridge Biotomography Centre (CBC). All scanned material was digitally segmented and rendered in three dimensions using VGStudio MAX 3.4.5 (Volume Graphics, Heidelberg, Germany). We segmented whole humeri into distinct regions of interest (ROIs) occupied by bone (high density), marrow (medium density) and air (lowest density) based on threshold grey value differences. For each humerus, bone volume (*V*_bone_), marrow volume (*V*_marrow_) and air volume (*V*_air_) were separately calculated using the voxel count of each ROI multiplied by voxel size in VGSTUDIO MAX 3.4.5. The ratio of humeral diameter (measured dorsoventrally) to scan resolution varied between 22 and 121. See electronic supplementary material for details of our segmentation approach, complete data on the grey value thresholds used for each modelled ROI, and our full volumetric dataset.

### (c) Quantifying pneumaticity

To quantify pneumaticity, we employed a three-dimensional air space proportion (ASP) measure, similar to that used by Moore [27]. However, unlike previous studies that use ASP as a quantifiable measure of pneumaticity, our method does not assume that the entire endocast of a pneumatized bone is filled with air (which implies the complete absence of bone marrow). Instead, by examining frozen bird specimens, we created three-dimensional models documenting the position and dimensions of air pockets present within a bone, as well as soft tissues occupying part or all of the internal bone cavity (which we define as ‘marrow’). This enables pneumaticity to be quantified in two ways: (i) calculating air space as a proportion of the total volume of an internal bone cavity (i.e. *V*_air_/*V*_air__+__marrow_), which we refer to as ASP_i_ and (ii) air space as a proportion of total bone volume (i.e. *V*_air_/*V*_air__+__marrow__+__bone_), which we refer to as ASP_t_. For additional discussion of ASP and interpretive caveats, see electronic supplementary material.

### (d) Comparative analyses

We used our data to test several established hypotheses regarding the evolution of postcranial pneumaticity in birds, specifically those relating to body size, diving ecology and bone thickness (bone amount). We carried out phylogenetic generalized least squares regression analyses (PGLS) [39] to evaluate the relationship of humeral internal air space with body mass, and the relationships of humeral bone volume—which is related to cortical bone thickness—with pneumaticity, body mass and/or an aquatic diving ecology. PGLS analyses were carried out using the *R* [40] package *caper* version 1.0.1 [41], with Pagel's *λ*, an estimator of phylogenetic signal in the relationship between variables [42], estimated using maximum likelihood. Our simplest models explain air volume and bone volume as a function of internal cavity volume (e.g. ‘air volume ∼ internal volume’), reflecting a ‘null hypothesis’ that air and bone volume simply scale with humeral size, and are independent of other factors, including ecological traits. The residuals from these simple models also provide estimates of allometric effects (in relation to humeral size) and correct for element size while avoiding the use of ratios or proportions [43]. We then added other variables to our models to test hypothesized explanations of this residual variation. These models evaluate the independent effects of additional variables on the scaling of air volume and bone volume. For example, the model ‘air volume ∼ internal volume + body mass' asks whether relative air volume scales with body mass, and the model ‘bone volume ∼ internal volume + diving’ asks whether relative bone volume varies depending on aquatic diving ecology. Our bone volume analyses included an evaluation of the independent effects of body mass, the presence/absence of pneumaticity and the presence/absence of aquatic diving ecology across *n* = 57 species. Air volume models could only be evaluated for the subset of birds exhibiting pneumatized humeri (*n* = 36 species), and only one diving bird in our sample had air in the humerus. Therefore, we could not analyse the effects of pneumatization or diving on air volume.

We performed model selection based on the Akaike information criterion for finite sample sizes (AICc) [44]. For our air volume models, our most complex model included internal volume and body mass as explanatory variables (i.e. ‘air volume ∼ internal volume + body mass'). For our bone volume analyses, our most complex model included internal volume, body mass, pneumaticity and diving as explanatory variables, as well as interactions between internal volume and pneumaticity, and internal volume and diving (i.e. ‘bone volume ∼ internal volume + body mass + diving + pneumaticity + internal volume × pneumaticity + internal volume × diving’). Interactions with body mass were not included because the resulting models were complex and difficult to interpret with respect to well-defined hypotheses. All volume measurements and body mass were log_10_-transformed prior to analyses.

### (e) Quantifying bone thickness

We used the residuals from our simplest bone volume PGLS model (bone volume ∼ internal volume) as an index of cortical bone thickness, which we refer to as the bone thickness index (BTI). Bone volume and internal humeral volume scale with humeral size, therefore, positive residuals from this model (and positive BTI numbers) represent relatively thicker humeral bone, and negative residuals from this model (and negative BTI numbers) represent relatively thinner humeral bone. Although BTI is not a direct measure of cortical bone thickness at any single point along a bone, it is proportional to a bone's average thickness, after correcting for allometry, across all possible sections of the bone.

### (f) Additional data collection

Information on aquatic diving habits was obtained from *Birds of the World* [45]. Body mass data were extracted from Dunning [46], except for *Fratercula arctica*, which was obtained from Lowther *et al*. [47].

To calculate whole bone (bulk) densities, we first estimated the total mass of each humerus, incorporating both bone and marrow. Avian humeral bone density was assumed to be 2.05 g cm^−3^, based on mean avian humeral density estimates from Dumont [48]. By multiplying this value by our bone volume measurements (mm^3^), we obtained an estimate of bone mass. Marrow density was assumed to be that of water (1 g cm^−3^ or, 0.001 g mm^−3^)—this assumption was based on previous calculations of marrow density which ranged from 0.9128 g cm^−3^–1.08 g cm^−3^ [49]. By multiplying assumed marrow density by our quantitative estimates of marrow volumes derived from CT scans (mm^3^), we obtained an estimate of marrow mass within a bone. Total humeral mass was then taken to be the sum of bone mass and marrow mass (assuming that air mass is negligible), and whole bone density was calculated as the estimated total humeral mass divided by the total bone volume (*V*_air__+__marrow__+__bone_).

## 3. Results

**Figure 1.**
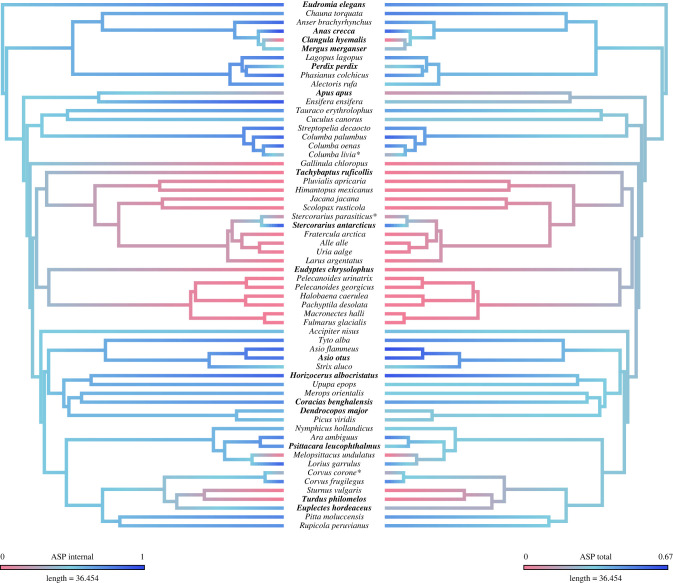
Time-scaled phylogenetic topology and pneumaticity scores for the species included in our study. Phylogeny follows Cooney *et al*. [50] and Prum *et al*. [51]. Branch colours are on a continuous scale and indicate the extent of humeral pneumaticity measured as ASP of the internal cavity (ASP_i_) (left) and ASP of the total bone volume (ASP_t_) (right). Apneumatic bones appear at the pink end of the scale, and pneumatic bones appear as shades of blue, depending on the extent of humeral pneumaticity. Species names in bold indicate those illustrated in electronic supplementary material, figure S3. Species marked with an asterisk (*) indicate suspected instances of incomplete PSP development (see discussion).

Of the 60 species included in our final analyses, 21 exhibited apneumatic humeri (entirely marrow-filled) and 39 exhibited pneumatized humeri. Of those that were pneumatized, our results illustrate a considerable amount of variation in the extent of that pneumaticity, both in terms of air space as a proportion of internal cavity volume (ASP_i_) and air space as a proportion of total bone volume (ASP_t_) (figure 1). ASP_i_ ranged from 29% in *Apus apus* to 98% in *Anas crecca*, with a mean of 74%, and ASP_t_ ranged from 11% in *Apus apus* to 66% in *Asio flammeus*, with a mean of 43%. For further information on ASP results, see electronic supplementary material.

### (a) Patterns of taxonomic variation

As the only member of Palaeognathae examined in this study, the tinamou *Eudromia elegans* represents the extrant sister taxon to the clade comprising all other investigated taxa. *E. elegans* exhibited a highly pneumatic humerus, calculated both as humeral ASP_i_ (83%) as well as ASP_t_ (49%), exceeding pneumaticity values of most of the remaining taxon sample. Among Neognathae, the large hypothesized waterbird clade Aequorlitornithes exhibited almost uniformly apneumatic humeri among species that we sampled, with the only exceptions being skuas (Charadriiformes: Stercorariidae), of which several representatives have been found to exhibit humeral pneumatization [52]. The humerus of the adult skua *Stercorarius antarcticus* was relatively highly pneumatized (ASP_i_ = 92%; ASP_t_ = 53%), illustrating a notable reversal to a highly pneumatized humerus within a large clade generally lacking humeral pneumaticity. Other instances of secondary humeral pneumatization arising among aequornithines unsampled in this study have also been noted previously in taxa such as frigatebirds (Fregatidae), boobies (Sulidae), pelicans (Pelecanidae) and the shoebill (*Balaeniceps rex*) [29,30]. Anseriformes (waterfowl) exhibited highly variable degrees of pneumatization, ranging from the entirely apneumatic humerus of the diving long-tailed duck *Clangula hyemalis* (ASP_i_ = 0%; ASP_t_ = 0%) to the highly pneumatized humerus of the dabbling Eurasian teal *Anas crecca* (ASP_i_ = 98%; ASP_t_ = 60%). Galliformes (landfowl), the sister taxon of Anseriformes, also exhibited a notable degree of variation in the extent of humeral pneumaticity, ranging from the grey partridge *Perdix perdix* (ASP_i_ = 50%; ASP_t_ = 30%) to the ring-necked pheasant *Phasianus colchicus* (ASP_i_ = 86%; ASP_t_ = 50%). We sampled multiple representatives of Psittaciformes (parrots), which generally exhibited highly pneumatized humeri (mean ASP_i_ of 75%), with the exception of budgerigars (*Melopsittacus undulatus*), which exhibit apneumatic humeri. We also sampled a number of Passeriformes and found that they too generally exhibited pneumatized humeri, although those of the thrush *Turdus philomelos* and its sister taxon in our dataset, the starling *Sturnus vulgaris*, were apneumatic (figure 1), documenting an apparent transition to apneumatic humeri within the passerine subclade Muscicapoidea [53]. Pigeons (Columbiformes) were found to exhibit relatively highly pneumatized humeri across the clade, with a mean ASP_i_ of 86.5% and a mean ASP_t_ of 53.4%. See electronic supplementary material for detailed discussion of patterns of taxonomic variation in skeletal pneumaticity.

### (b) Extent of body mass reduction due to pneumatization

**Figure 2.**
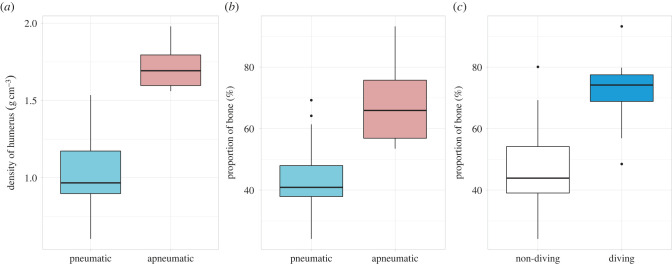
Box and whisker plots showing (*a*) variation in bulk density in pneumatic and apneumatic humeri, (*b*) proportion of bone volume relative to total humeral volume (%) in pneumatic and apneumatic humeri, and (*c*) proportion of bone volume relative to total humeral volume (%) in taxa exhibiting non-diving and aquatic diving ecologies. Bold lines on box plots represent the median. Upper whiskers correspond to data within 1.5 times the interquartile range over the 75^th^ percentile, lower whiskers correspond to data within 1.5 times the interquartile range under the 25^th^ percentile.

Pneumatic bones are expected to be less dense than apneumatic bones due to the replacement of comparatively heavy marrow by air. However, because birds exhibit variation in both bone and soft tissue volumes across the avian crown group, we were interested in estimating the extent of whole bone (bulk) density reduction conferred by humeral pneumatization. We found that pneumatic humeri varied in density between 0.60 and 1.53 g cm^−3^, with a mean of 1.02 g cm^−3^, while apneumatic humeri varied in density between 1.56 and 1.98 g cm^−3^, with a mean of 1.71 g cm^−3^. We observed a much greater degree of density variation among pneumatic humeri than among apneumatic humeri. Although there was no overlap in density ranges between pneumatic and apneumatic humeri, the densest pneumatic humeri (e.g. *Apus apus*, *Ensifera ensifera* and *Euplectes hordeaceus*) approached the densities of the least-dense apneumatic humeri (e.g. *Jacana jacana*, *Himantopus mexicanus* and *Pluvialis apricaria*) (figure 2*a*; electronic supplementary material, table S1).

**Figure 3.**
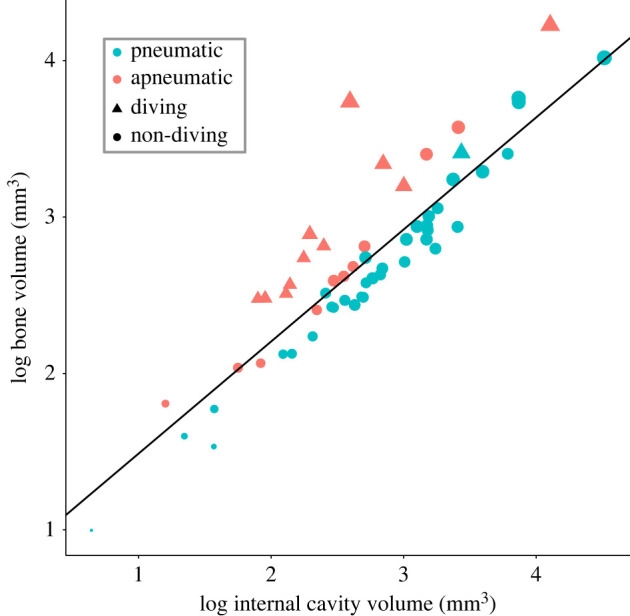
Scatter plot of the ‘null hypothesis’ model for the bone volume model family of our PGLS analyses. Data points falling above the regression line (i.e. those with positive residual variation) are representative of species with relatively thicker humeri, and those that fall below the regression line (i.e. those with negative residual variation) are representative of species with relatively thinner humeri—this is a direct reflection of our BTI. Colours represent apneumatic (pink) and pneumatic (blue) humeri. Data point shape reflects taxa exhibiting specialized aquatic diving (triangles) and non-diving (circles) ecologies. Data point size corresponds to log10-transformed body mass (g), with larger-bodied birds represented by increasingly larger data points (values range from 1.05 to 3.65).

Apneumatic humeri exhibited relatively greater bone volumes on average (i.e. thicker bones as assessed by our BTI; mean apneumatic BTI = + 0.27), compared with pneumatic humeri (mean pneumatic BTI = −0.11) (figure 3). BTI of pneumatic humeri varied between −0.36 (the Asian green bee-eater *Merops orientalis*) and +0.22 (the pink-footed goose *Anser brachyrhynchus*), whereas values for apneumatic humeri varied between −0.08 (the song thrush *Turdus philomelos*) and +1.11 (the Macaroni penguin *Eudyptes chrysolophus*). However, the Macaroni penguin (BTI +1.11) is a clear outlier in this dataset; the second-highest BTI in our dataset was much lower (+0.53 in the common murre *Uria aalge*). Of the 57 species included in our analyses, 12 are specialized aquatic diving (non-specific) foragers (see electronic supplementary material, table S1). Of those 12 diving species, only the anseriform common merganser *Mergus merganser* exhibited pneumatized humeri (although this is one of the few representatives of the diving anseriform subclade Mergini known to exhibit humeral pneumatization [20]). Humeri of specialized diving birds had relatively thicker bone on average (mean BTI = + 0.41) compared with those of non-divers (mean BTI = −0.07). Regardless of pneumatic state, all specialized diving birds exhibited humeri with a positive BTI, indicating that they all have relatively thick humeri (figure 3).

### (c) Testing functional hypotheses regarding the evolution of humeral pneumatization

Among PGLS regression models constructed to evaluate variation in air volume, the best-supported model according to AICc is our simplest model, which explains air volume as a function of internal cavity volume (AICc weight = 0.687; table 1). This supports a hypothesis that air volume simply scales with humeral size, lacking independent effects of body mass. The coefficient of internal volume in this analysis indicates weak positive allometry (coefficient = 1.044, compared to 1.0 expected under isometry; s.e. = 0.023, *p* < 0.0001; table 1), and the relationship is highly explanatory (*R*^2^ = 0.984). The lack of influence of body mass on humeral air volume is corroborated by the fact that the independent effect of body mass is non-significant in the model ‘air volume ∼ internal volume + body mass’ (table 1).

**Table 1.** Phylogenetic regressions comparing explanations of humeral air volume variation. Only the best model according to AICc (in italics) and models with non-negligible AICc weights (greater than 1/8 of the best model) are shown. Results for the full set of models are shown in the electronic supplementary material, table S3. Abbreviations: AICc, Akaike Information Criterion for finite sample sizes; AICc Wt, AICc weight; *R*^2^, coefficient of determination; lambda (*λ*), Pagel's lambda; s.e., standard error.

PGLS model syntax	*n*	AICc	AICc Wt	*R*^2^	lambda	variable	estimate	s.e.	*t*-value	Pr(>|*t*|)
*air volume* ∼ *internal volume*	36	−58.94	0.687	0.984	0	(intercept)	−0.27	0.067	−4.045	<0.0001
						internal volume	1.044	0.023	45.777	<0.0001
air volume ∼ internal volume + body mass	36	−57.36	0.313	0.983	0	(intercept)	−0.245	0.073	−3.366	0.002
						internal volume	1.093	0.061	17.978	<0.0001
						body mass	−0.068	0.078	−0.867	0.392

PGLS regression analyses were also carried out to investigate variation in bone volume, while controlling for allometry (internal cavity volume). The simplest model (bone volume ∼ internal volume) shows a positive relationship between bone volume and internal cavity volume, which is as expected if both tend to increase with increasing humeral size. The residual variation from this ‘null hypothesis’ model represents differences in relative bone volume, independent of internal cavity volume, and are therefore used here as a measure of bone cortical thickness—we would expect thicker bones to fall above the regression line, and thinner bones to fall below it (figure 3).

The best-supported model to explain variation in humeral bone volume includes internal volume, body mass and categorical variables describing the presence/ absence of diving and the presence/ absence of pneumaticity (AICc weight = 0.428; table 2). The coefficients of this model are highly significant (*p* < 0.0001 for all variables; table 2) and the model is highly explanatory (*R*^2^ = 0.972). This model contains two size-related coefficients, that of internal volume (coefficient = 0.5, s.e. = 0.047, *p* < 0.0001; table 2) and that of body mass (coefficient = 0.428, s.e. = 0.059, *p* < 0.0001; table 2). The summed coefficients (= 0.928) indicate weak negative allometry of bone volume (compared to a coefficient of 1.0 expected under isometry). These results demonstrate that the humeri of larger-bodied bird species exhibit proportionally thinner bone relative to the internal cavity than smaller species, independent of the effects of pneumaticity or aquatic diving ecologies. The coefficient of our pneumaticity variable in this model (coefficient = −0.193, s.e. = 0.037, *p* < 0.0001; table 2) indicates that the presence of pneumaticity in the humerus has a significant negative effect on relative bone volume, meaning that pneumatic humeri have proportionally less bone volume than apneumatic humeri (figure 3), independent of variation in internal space and independent of the effects of body mass and aquatic diving ecologies. This suggests that increases in the extent of humeral pneumatization are achieved in part by replacing bone with air, and not only by replacing marrow with air. The coefficient of our diving variable in this model (coefficient = 0.174, s.e. = 0.045, *p* < 0.0001; table 2) indicates that the presence of aquatic diving ecologies has a significant positive effect on relative humeral bone volume, meaning that humeri of species with aquatic diving ecologies have proportionally more bone volume (and therefore thicker bone) than those belonging to species with non-aquatic diving ecologies (figure 3), independent of variation in internal space and independent of the effects of body mass and presence of pneumaticity.

**Table 2.** Phylogenetic regressions comparing explanations of humeral bone volume variation. Only the best model according to AICc (in italics), and models with non-negligible AICc weights (greater than 1/8 of the best model) are shown. Our simplest ‘null hypothesis’ model (bone volume ∼ internal volume) is also shown for comparison. Results for the full set of models are shown in the electronic supplementary material, table S4. Abbreviations are the same as for table 1.

PGLS model syntax	*n*	AICc	AICc Wt	*R*^2^	lambda	variable	estimate	s.e.	*t*-value	Pr(>|*t*|)
*bone volume* ∼ *internal volume + body mass + diving + pneumaticity*	57	−92.1	0.428	0.972	0	(intercept)	0.434	0.063	6.841	<0.0001
						internal volume	0.5	0.047	10.733	<0.0001
						body mass	0.428	0.059	7.223	<0.0001
						diving_TRUE	0.174	0.045	3.833	<0.0001
						pneumaticity_TRUE	−0.193	0.037	−5.235	<0.0001
bone volume ∼ internal volume + body mass + diving + pneumaticity + internal volume: pneumaticity	57	−90.33	0.177	0.972	0	(intercept)	0.373	0.099	3.779	<0.0001
						body mass	0.428	0.06	7.186	<0.0001
						diving_TRUE	0.173	0.045	3.802	<0.0001
						internal volume	0.526	0.056	9.31	<0.0001
						pneumaticity_TRUE	−0.104	0.115	−0.903	0.371
						internal volume:Pneumaticity_TRUE	−0.035	0.043	−0.812	0.421
bone volume ∼ internal volume + body mass + pneumaticity	57	−90.01	0.151	0.964	0.82	(intercept)	0.435	0.08	5.451	<0.0001
						internal volume	0.426	0.049	8.684	<0.0001
						body mass	0.507	0.061	8.252	<0.0001
						pneumaticity_TRUE	−0.165	0.041	−3.978	<0.0001
bone volume ∼ internal volume + body mass + diving + pneumaticity + internal volume: diving	57	−89.87	0.141	0.972	0	(intercept)	0.421	0.069	6.13	<0.0001
						body mass	0.431	0.06	7.188	<0.0001
						pneumaticity_TRUE	−0.192	0.037	−5.161	<0.0001
						internal volume	0.502	0.047	10.649	<0.0001
						diving_TRUE	0.24	0.142	1.697	0.096
						internal volume:diving_TRUE	−0.026	0.052	−0.497	0.621
bone volume ∼ internal volume + body mass + diving + pneumaticity + internal volume: pneumaticity + internal volume: diving	57	−89.25	0.103	0.972	0	(intercept)	0.277	0.128	2.164	0.035
						body mass	0.433	0.059	7.281	<0.0001
						internal volume	0.558	0.063	8.902	<0.0001
						diving_TRUE	0.36	0.167	2.159	0.036
						pneumaticity_TRUE	−0.014	0.139	−0.101	0.92
						internal volume:diving_TRUE	−0.072	0.062	−1.167	0.249
						internal volume:pneumaticity_TRUE	−0.069	0.052	−1.332	0.189
bone volume ∼ internal volume	57	−20.2	0	0.878	0.92	(intercept)	0.771	0.128	6.048	<0.0001
						internal volume	0.716	0.036	20.097	<0.0001

## 4. Discussion

This study quantitatively documents variation in the extent of humeral pneumaticity across the avian crown group for the first time. We demonstrate considerable variation in the extent of humeral pneumaticity among major avian subclades (figure 1) and illustrate that extensive humeral pneumatization is widespread across the avian crown group. The presence of a highly pneumatic humerus in palaeognaths (represented in our sample by the tinamou *Eudromia elegans*; ASP_i_ = 83%; ASP_t_ = 49%), as well as in many galloanserans and neoavians, indicates that the last common ancestor of crown birds most likely exhibited an extensively pneumatized humerus.

### (a) Relationship between extent of pneumaticity, humeral size and body size

We found strong evidence for weak positive allometry of humeral air volume with humeral size (internal cavity volume). This suggests that larger birds have proportionally more air relative to marrow in the humerus. We found no evidence to suggest that increasing relative body mass correlates with any further increase in humeral air volume (after accounting for increasing humeral size). Previous studies have also reported an association between increased body size and number of pneumatic elements across the skeleton [20,26,29,30], although support for this relationship is variable, with studies using phylogenetic comparative methods often failing to provide support for this association [35]. Collectively, these findings suggest that larger bodied birds may exhibit greater numbers of pneumatized bones overall, and also that birds with larger humeri (which in general are larger bodied birds) have a greater proportion of air replacing marrow in the internal cavity of their pneumatic elements. Future studies will be necessary to robustly evaluate this hypothesis by exploring variation in air volume within other bones of the skeleton, and among different avian subclades.

### (b) Relationships between pneumatization and other osteological parameters

Previous authors have demonstrated that apneumatic bones exhibit increased cortical bone thickness compared to pneumatized bones [28,31,32,37]. Our findings corroborate these results—our PGLS analyses of the independent effects of pneumaticity on humeral bone volume, after correcting for humeral internal cavity volume, suggest that apneumatic humeri exhibit proportionally more bone volume compared to pneumatized humeri.

Foraging ecology and locomotor mode have previously been identified as key factors influencing the extent of skeletal pneumatization [20,26,29,30,35]. Notably, these studies have found a general decrease in the extent of pneumaticity across the skeleton in aquatic diving taxa. Aquatic diving ecologies have also previously been associated with an increase in skeletal density as a result of increased bone thickness, which has been interpreted as an energy-saving adaptation for maintaining neutral buoyancy underwater [31,36–38]. Our findings corroborate this hypothesis—our PGLS analyses on the independent effects of aquatic diving ecologies on humeral bone volume, after correcting for humeral internal cavity volume, and taking into account the independent effects of pneumaticity and body mass, suggest that humeri belonging to species with aquatic diving ecologies exhibit proportionally thicker bone compared to humeri belonging to non-divers. In our study, birds with diving ecologies, such as penguins (Aequornithes: Spheniscidae), auks (Charadriiformes: Alcidae) and numerous petrels and kin (Procellariiformes: Procellariidae), exhibit some of the highest observed proportions of humeral bone volume, as well as the thickest bone according to BTI (figure 3; electronic supplementary material, table S1). The penguin *Eudyptes chrysolophus* exhibited by far the greatest proportion of bone (93%) as well as relative bone thickness (BTI +1.11) in our sample—probably because penguin flightlessness has allowed for an increase in mass through the replacement of marrow volume in the internal cavity with heavier cortical bone to maintain neutral buoyancy [31,36–38]. Volant birds that also engage in aquatic pursuit diving, such as the common murre (*Uria aalge*) and other extant auks (Alcidae) remain subject to a trade-off between the energetic benefits of a heavier skeleton (beneficial for aquatic diving) and a light skeleton (beneficial for aerial flight; [54]). In our dataset, no representatives of Alcidae exceeded a humeral bone volume proportion of 80% (the Atlantic puffin *Fratercula arctica*), nor a BTI greater than +0.53 (the common murre *U. aalge*), considerably lower than the aforementioned values in the penguin. Maintaining the ability to fly therefore limits the extent to which marrow volume in the internal cavity can be replaced by heavier cortical bone in aquatic pursuit divers, with volant pursuit divers such as auks probably approaching a limit as to what is functionally feasible.

Among flighted birds, the conservation of relative humeral bone thickness within a relatively small range (BTI −0.36 to +0.53) across the avian crown group may be indicative of a functional or biomechanical constraint. For example, in pneumatized bones, a trade-off may exist whereby selection for body mass reduction drives decreases in humeral bone thickness, but is balanced by selection for maintaining enough bone mass to enable torsional resistance of the humerus during flight. This type of constraint might also lead to changes in bone shape [27], for example, in instances where mass reduction is under strong selection. This hypothesis requires further study with regard to its relevance in the humerus and in other long bones.

### (c) Post-fledging progression of postcranial skeletal pneumaticity

**Figure 4.**
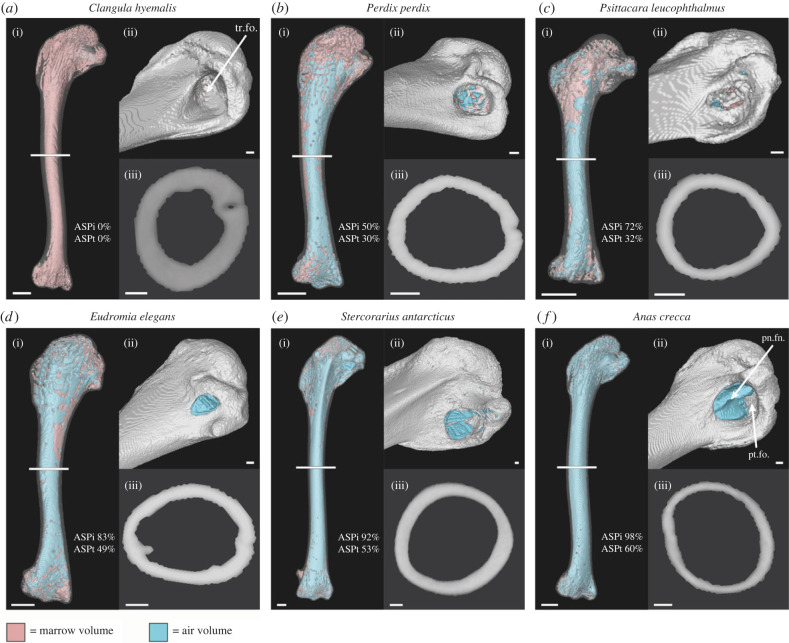
Taxa exemplifying the range of variation in the extent of humeral pneumaticity across crown bird phylogeny, as calculated by ASP_i_ and ASP_t_. Left humerus of (*a*) long-tailed duck *Clangula hyemalis*, (*b*) grey partridge *Perdix perdix*, (*c*) white-eyed parakeet *Psittacara leucophthalmus*, (*d*) elegant crested tinamou *Eudromia elegans* and (*e*) brown skua *Stercorarius antarcticus*, and mirrored right humerus of (*f*) Eurasian teal *Anas crecca*. (i) Caudal view, with bone set at 75% transparency and the internal cavity displaying the modelled endocast volumes of marrow (pink) and air (blue). (ii) Close-up image of the (pneumo-)tricipital fossa. (iii) Cross-section of the midshaft of the diaphysis, used for humeral diameter measurements. White bar in (i) indicates position of the cross-section in (iii). Scale bars represent 5 mm (i) and 1 mm (ii, iii). Abbreviations: pn.fn., pneumatic foramen; pt.fo., pneumotricipital fossa(e); tr.fo., tricipital fossa(e). Note: we restrict use the term ‘pneumotricipital fossa’ to describing the tricipital fossa in taxa with confirmed pneumatized humeri. For taxa with apneumatic humeri we use the term ‘tricipital fossa’.

In their study of pneumaticity in avian long bones, Cubo & Casinos [32] used a method of direct observation through the diaphysis (shaft) of the bones to assess whether or not they were air-filled (pneumatic) or marrow-filled (apneumatic). They stated that ‘no ambiguous state was found’. We find the situation to be more complex. For the most part, our findings show that apneumatic humeri are entirely marrow-filled (although, see discussion on potential false positive interpretations of pneumaticity in the electronic supplementary material), and that pneumatized humeri have air that enters through the pneumatic foramen at the proximal end of the bone and extends at least to the distal end of the diaphysis (figure 4). Our three-dimensional models show that any soft tissues that are present in a pneumatic bone are generally either present mainly in the epiphyses, or scattered along the inside wall of the diaphysis. With the exception of three specimens, we did not observe large masses of soft tissue within the bone cavities of pneumatized humeri. These three unusual pneumatized specimens, however, exhibited varying amounts of marrow within the bone cavity, at the distal end of the diaphysis. These specimens, belonging to the feral rock dove *Columba livia* (Columbidae), parasitic jaeger *Stercorarius parasiticus* (Charadriiformes: Stercorariidae) and carrion crow *Corvus corone* (Passeriformes: Corvidae), have potentially ‘ambiguous’ states of humeral pneumatization, exhibiting partially air-filled humeri, with air entering the bone through the pneumatic foramen at the pneumotricipital fossa, but limited to the proximal end of the bone (electronic supplementary material, figure S2). This partly air-filled and partly marrow-filled state was present in both humeri in each of the specimens. Considering that the process of pneumatization occurs post-hatching, with pneumatic diverticula invading the bone through pneumatic foramina and gradually extending through the bone, replacing marrow with air [8,22,24,55], we speculate that these three specimens all represent immature birds that would have later lost this marrow within the humerus. Moreover, the sister taxa of these three species in our dataset all exhibit highly pneumatized humeri (figure 1; electronic supplementary material, figure S2). Although the acquisition of PSP is known to take place post-hatching, the timeline by which skeletal pneumatization arises in different bird species remains relatively unstudied [24] and represents an intriguing area for future research. All of the specimens in question in this study had fledged and reached their mature adult sizes, although the plumage of the parastic jaeger (*Stercorarius parasiticus*) showed that it was a first-year bird. If all of these specimens are immature, it suggests that the full extent of PSP development is sometimes, if not always, completed post-fledging, at least for the humerus. Interestingly, an early study on avian PSP similarly observed that the humeri of some young, yet fully-fledged hawks, owls and magpies, which all exhibit humeral pneumatization, were marrow-filled and did not exhibit a pneumatic foramen [56]. To our knowledge, no studies published since have further investigated this phenomenon. Although our speculations are based on observations from only three specimens, these species are distantly related to one another (figure 1), so post-fledging PSP completion could be a widespread phenomenon across the avian crown group, at least among relatively altricial taxa [57]. Future studies examining the development of pneumatic diverticula throughout avian ontogeny will be necessary to evaluate this hypothesis.

## 5. Conclusion

We present the first quantitative estimates of the true extent of pneumaticity in the humerus of extant birds, taking into account soft tissues present within their internal cavities. Our results show that humeral pneumaticity is phylogenetically widespread, and probably ancestral for the avian crown group. Moreover, both the presence and extent of humeral pneumaticity vary considerably among extant birds, with foraging ecology and locomotor behaviour (e.g. aquatic diving) linked to variation in these parameters.

Our findings indicate that previous studies that have applied quantitative ASP measurements to assess skeletal pneumaticity have systematically overestimated the proportion of bone volume occupied by air as a result of disregarding the presence of soft tissues within pneumatized bird bones. This will have implications for previously published estimates of avian bulk density and body mass, which could in turn have an effect on any hypotheses linking these factors to the evolution of skeletal pneumaticity. Our work illustrates the importance of studying fresh specimens with soft tissues intact as opposed to skeletonized specimens when attempting to quantify the extent of skeletal pneumaticity, which will in turn enable better constrained inferences regarding the origins and evolution of this key avian feature.

Our results add to the body of work investigating the extent of PSP in crown birds [20,26,29,30,35] and lay the groundwork for future studies that attempt to further categorize the true extent of pneumatization of specific skeletal elements. Sampling additional skeletal elements will enable variation in skeletal pneumaticity across entire skeletons to be assessed, and sampling a broader phylogenetic range of taxa—particularly those that are presently undersampled such as passerines and non-aequolitornithine neoavians—will be crucial for evaluating existing hypotheses regarding evolutionary links between the extent of PSP and factors such as body size and avian ecology.

## Data Availability

All data are presented in the electronic supplementary material. Scan data are openly accessible from MorphoSource (https://www.morphosource.org/projects/000489577?locale=en; project ID 000489577). The data are provided in the electronic supplementary material [58].
